# Single-Stage Orbital Socket Reconstruction Using the Oversized Dermis Fat Graft and the 22 mm Silicone Orbital Implant after an Extended Enucleation

**DOI:** 10.1155/2018/8954193

**Published:** 2018-12-04

**Authors:** Biljana Kuzmanović Elabjer, Mladen Bušić, Daliborka Miletić, Mirjana Bjeloš, Borna Šarić, Damir Bosnar

**Affiliations:** University Eye Clinic, Faculty of Dental Medicine and Health Care Osijek, Faculty of Medicine Osijek, University Josip Juraj Strossmayer in Osijek, University Hospital “Sveti Duh”, Zagreb, Croatia

## Abstract

We would like to present a surgical technique of orbital socket reconstruction using oversized dermis fat graft and 22 mm silicone orbital implant in a single-stage after extended enucleation in two patients with massive local recurrence of anteriorly located choroidal melanoma previously treated with endoresection. Orbital tissues en bloc were removed leaving conjunctival lining only at the fornices. Simultaneously, the 22 mm silicone sphere was implanted deeply into the orbit and covered with the oversized dermis fat graft of 30 mm in height and 35 mm in length with 20 mm of the fat thickness. The graft was sutured to the residual forniceal conjunctiva with interrupted 6/0 absorbable sutures overlapping conjunctiva with the graft edge for 2 mm to facilitate the epithelization. Epithelization was completed in two months, leaving well-formed fornices with good fitting of the prosthesis. The key point of orbital socket reconstruction after extended enucleation is to restore conjunctival lining prior to volume. Thus, whenever facing a massive volume and conjunctival lining loss, simultaneous insertion of the 22 mm silicone sphere deep into the orbit combined with oversized dermis fat graft is, in our opinion, the method of choice. It proved to be safe and effective with favourable long-term results.

## 1. Introduction

Dermis fat graft has been proved to be an effective method for orbital reconstruction after enucleation and evisceration, as either primary or secondary procedure [1–3]. It provides simultaneously volume augmentation as well as a scaffold for conjunctival overgrowth [4].

Local tumour recurrence after brachytherapy or endoresection of uveal melanoma is mainly treated with enucleation [5, 6]. When a large part of the bulbar conjunctiva is infiltrated with tumour an extended enucleation is an option. A resulting devastating loss of both lining and volume raises an issue of restoring orbital architecture to create fairly natural cosmesis, preferably, through a single-stage procedure. Failure to compensate for the volume can lead to postenucleation socket syndrome. Orbital implant alone will not compensate for the entire socket volume thus resulting in migration and expulsion [4]. Furthermore, massive conjunctival loss leaves no space to embed the eye prosthesis. Two major advantages place the perfectly biocompatible dermis fat as the “must have” implant. First, the fat volume loads the orbital socket and second it takes the epithelial characteristics of the conjunctiva during the integration process [7]. However, in the first six months estimated atrophy of the dermis fat graft reaches 25-35% in the recommended size of the graft that has an anterior surface diameter between 20 and 24 mm and a thickness of 20 mm [7]. Thus, the question of the optimal dermis fat graft size still remains an open dilemma.

The aim of this paper is to present a surgical technique of orbital socket reconstruction after extended enucleation in a single stage by combining the above-mentioned options and taking the best of each, i.e., using the oversized dermis fat graft with 22 mm silicone orbital implant. An extensive literature search using Medline, Scopus, and WoS demonstrated no report on the subject.

## 2. Case Report

**Figure 1:**
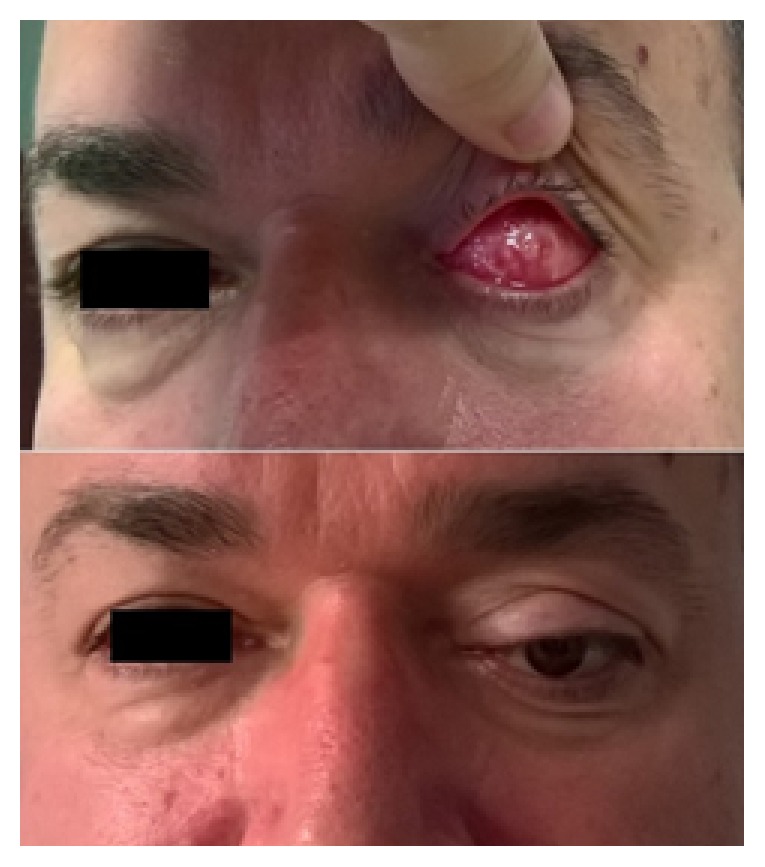
50-year-old Caucasian male patient 4 years after the procedure.

**Figure 2:**
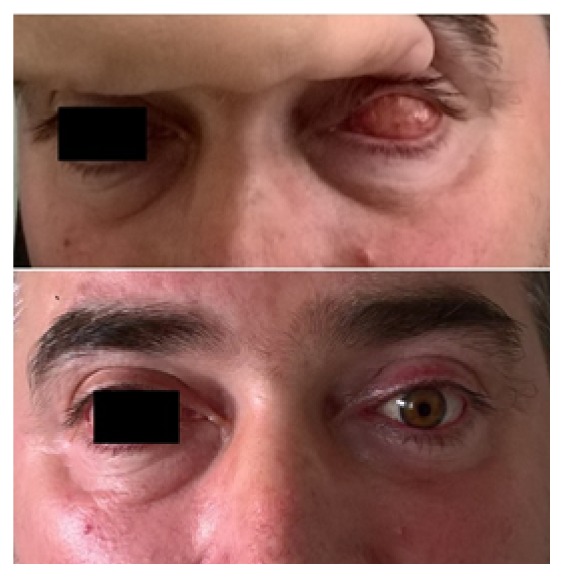
44-year-old Caucasian male patient 8 months after the procedure.

In January 2013, a 50-year-old Caucasian male patient underwent, without an adjunctive brachytherapy, endoresection of anteriorly located spindle cell choroidal melanoma. In December 2014, multiple pigmented scleral lesions were found on the same eye, with the outermost lesion located 5 mm away from the limbus. The second patient, 44-year-old Caucasian male, underwent the same procedure for the anteriorly located mixed cell choroidal melanoma, in July 2011. Six years later a massive recurrence of the tumour was infiltrating the anterior eye segment with extraocular limbal extension. Both patients underwent an extended enucleation with removal of almost all orbital tissues en bloc including the eye, anterior portion of the extraocular muscles, and long section of the optic nerve. Antiseptic douching of the eye with 10% povidone-iodine was performed preoperatively. Conjunctiva was opened at the fornices, so that the entire bulbar conjunctiva could have been removed with the eye en bloc, avoiding manipulation of the tumour-infiltrated areas. Dislocating the eye out, the extraocular muscles were cut as far posterior as possible. Optic nerve was severed approximately 10 mm from the eyeball. Silicone sphere of 22 mm (FCI Ophthalmics) was implanted deeply into the orbit through a glide made from the thumb of a sterile polythene glove [8]. During the same procedure, dermis fat graft, harvested from the left suprapubic area, was used to cover the implant [2]. The size of the graft was 30 mm in height and 35 mm in length with 20 mm of the fat thickness. Interrupted 6/0 absorbable sutures were used to fixate the graft to the residual conjunctiva at the fornices taking care of the fact that the conjunctiva overlaps the edge of the graft by two millimetres. At the end of the surgery, silicone conformer was inserted and kept for the entire time of the graft epithelization. Broad-spectrum topical antibiotic prophylaxis (tobramycin drops qid) was applied for seven postoperative days. Epithelisation of the dermis fat graft was completed in two months, leaving well-formed fornices with good fitting of the prosthesis (Figures 1 and 2). A small conjunctival granuloma appeared two months postoperatively in the first patient and was managed by simple surgical intervention. So far, the first patient has been followed for four years and the second for eight months, without experiencing any major complication [9, 10].

## 3. Discussion

Simultaneous secondary orbital implantation and dermis fat graft placement for exposed porous implants with significant conjunctival insufficiency has been described [11]. To the best of our knowledge, simultaneous usage of the silicone orbital implant of 22 mm and the oversized dermis fat graft for orbital socket reconstruction after an extended enucleation has not been reported so far. The key point of orbital socket reconstruction after extended enucleation needs to be emphasized: the conjunctival lining needs to be reconstructed before the volume restoration. Thus, usage of only a silicone implant is inadequate. Although dermis fat graft remodels the lining perfectly, in adults the volume restitution comes into question due to unpredictable fat reabsorption [12].

Local recurrence of choroidal melanoma may result in extraocular extension and massive bulbar conjunctiva involvement [13]. Orbital exenteration for uveal melanomas with extrascleral spread is considered only in presence of gross orbital tumour extension [13]. Even then, in most instances the eyelid-sparing exenteration is performed [14]. In both our patients there was no orbital invasion, only anterior bulbar conjunctival involvement that spared fornices, enabling complete removal of the tumour through extended enucleation. Because of the risk of involvement of the extraocular muscle tendons underlying the infiltrated conjunctiva, the standard enucleation was not a method of choice. In an adult, the orbital volume is 30 ml. Enucleation of the eyeball reduces it by 6-7 ml and associated-tissue loss by additional 1-2 ml [15]. In the extended enucleation, the volume deficiency is even more pronounced. Instead of adding an orbital implant to the dermis fat graft in the second surgery, the authors insisted on the single-stage procedure having in mind the fact that fat layer contraction of the graft in absence of an implant would have prevented fitting the best radius sphere in second stage of the surgery. We recommend adding the oversized dermis fat graft to the silicone orbital implant of 22 mm, with a volume of ≈ 5.5 ml, in a single procedure. The need for volume must be balanced against the risk of extrusion if the implant is too large, although this complication is less common in a primary surgery [16]. The ideal volume replacement is spherical orbital implant of 21 to 22 mm diameter [17]. After massive implant extrusion, dermis fat graft can be combined with orbital implant insertion concomitantly or in the second stage [2]. In case of the exposed or infected orbital implant, simultaneous insertion of a new orbital implant with autologous dermis-graft may cause the graft to fail because no vascularized tissue lies underneath [12]. Secondary insertion of the implant after preceding dermis fat graft is here a preferred method [18].

After assessing fat panicle thickness at the left suprapubic area, it was chosen as a harvest point for the dermis fat graft since it is not under pressure and contains only lanugo hears and the scar is easily hidden under swimwear [2, 7]. The existent recommendations for the size of dermis fat graft have been adjusted in our patients to avoid undercorrection and central necrosis due to compression and ischemia in oversized graft [7]. Apart from adding volume and vascular support, fat and dermis act as a temporary biologic dressing [7]. The author's technique of suturing the graft involves the conjunctiva overlapping the edge of the dermis by two millimetres that, in our experience, facilitates the epithelization. Once epithelized, dermis takes the role of bulbar conjunctiva. Together with preserved fornices, it enables good fitting of the prosthesis, which adds two millilitres of volume to the orbit.

Nonintegrated orbital implant was used as the author's personal preference. Both integrated and nonintegrated implants are well tolerated with low complication rates and with no difference in motility, implant extrusion, removal, or need for secondary procedures [18, 19]. Silicone orbital implant had the lowest overall number of complications even as a secondary implant in the large study of Shoamanesh et al. [9]. The wrapping of an implant is done in order to allow attachment of the extraocular muscles and to prevent exposure. Since anterior parts of the muscles are removed in extended enucleation, wrapping the silicone implant is not necessary. Furthermore, dermis fat graft itself serves as a barrier against implant extrusion and migration.

Motility of prosthesis can be enhanced by deep conjunctival fornices and large orbital implants. In addition, placement of the implant deep in the orbit is mandatory [8]. The authors honoured these postulates, and even after extended enucleation with only posterior remnants of the muscles preserved, a certain level of motility of the prosthesis is achieved. This surgical technique proved to be successful in both our patients.

The key message of this report outweighs its limitation such as defined number of cases. The authors encourage colleagues to broaden indications for an extended enucleation and, in case of one, to embrace simultaneous usage of orbital implant and oversized dermis-fat graft as a primary procedure of the reconstruction.
